# Barriers associated with pulse and plant-based meat alternative consumption across sociodemographic groups: a Capability, Opportunity, Motivation, Behaviour model approach

**DOI:** 10.3389/fnut.2023.1186165

**Published:** 2023-08-29

**Authors:** Sini Kuosmanen, Mari Niva, Anne-Maria Pajari, Kirsi Korhonen, Toivo Muilu, Hanna Konttinen

**Affiliations:** ^1^Department of Food and Nutrition, University of Helsinki, Helsinki, Finland; ^2^Department of Economics and Management, University of Helsinki, Helsinki, Finland; ^3^Natural Resources Institute Finland, Oulu, Finland; ^4^Social Psychology, Faculty of Social Sciences, University of Helsinki, Helsinki, Finland

**Keywords:** plant-based diet, alternative protein, legume, socioeconomic differences, COM-B

## Abstract

**Introduction:**

To enhance environmental sustainability and food security, there should be a change in dietary protein consumption. It is suggested that meat consumption should be reduced and that the currently low consumption of pulses and other plant-based proteins should increase. We aimed to examine (1) how sociodemographic factors and perceived barriers are associated with self-reported current and perceived future pulse and other plant-based meat alternative (PBMA) consumption and (2) how sociodemographic factors relate to perceived barriers.

**Methods:**

Participants were 18–75 year-old Finnish adults (*n* = 1,000). Multivariable logistic regression was used as the main analysis technique. The results were interpreted by employing the Capability, Opportunity, Motivation, Behaviour (COM-B) model.

**Results:**

Pulses were consumed more often than PBMAs and lower education level and financial strain were associated with more infrequent pulse and PBMA use. The most common perceived barriers for pulse consumption were unfamiliarity, expensive price, and unpleasant taste, which can be interpreted to represent the capability, opportunity and motivation components of the COM-B model, respectively. Women, the young, and financially strained perceived more barriers limiting their pulse consumption than others.

**Discussion:**

To increase plant-based food consumption, it is important that tasty, easy to use and affordable plant-based foods are available for all. Additionally, we suggest that food services should be encouraged to increase the use of pulses in their dishes and that capabilities, opportunities and motivations are taken into account in intervention measures advancing plant protein consumption.

## 1. Introduction

As the world faces new and recurring challenges, such as the climate change and the ever-growing global population, it is more important than ever to rethink the sustainability of our dietary and food production practices. Current high level of meat production is regarded as environmentally unsustainable (1), and plant-based proteins have generally smaller environmental footprint than animal proteins (2, 3). Despite this, meat consumption has increased significantly globally in the last decades (4). Cultivating more sustainable protein sources, such as pulses, can contribute to mitigating climate change (5), but it is also noteworthy to recognize that pulse cultivation is not unproblematic either [e.g., (6)]. Despite the shortcomings of pulse cultivation, it is widely acknowledged that there should be a transformation in dietary protein sources: for instance, in 2019 the EAT-Lancet Commission proposed a substantial increase in pulse consumption while reducing meat consumption to ensure global food production and healthy diets within planetary boundaries (7).

Meat has been regarded as a central ingredient in main meals especially in Western diets (8), while the consumption of pulses is generally low; for example, in Finland about 140 grams weekly (9), which is well below the recommended 525 grams weekly as suggested by the EAT-Lancet Commission (7). One reason behind this is that different sociodemographic groups often experience different kinds of barriers related to eating pulses – a relationship that needs to be better understood to increase the environmental sustainability of food consumption. Furthermore, a recent review on meat alternatives by Onwezen et al. (10) suggests that more research on pulse and plant-based meat alternative (PBMA) consumption is needed, as only 18 of the 91 articles reviewed concerned pulses or PBMAs.

In this paper, we examine how the barriers and sociodemographic factors are associated with current and future pulse and PBMA consumption, as well as how sociodemographic factors relate to these barriers. The conceptualization of the Capability, Opportunity, Motivation, Behaviour (COM-B) model (11) is applied in examining the barriers affecting plant-based food consumption. In addition, sociodemographic characteristics such as gender, age, education and income level are taken into account in the analysis, as they may be reflected on an individual’s capabilities, opportunities and motivations to consume plant-based foods.

## 2. Applying the COM-B model in the context of plant-based eating across sociodemographic groups

### 2.1. Sociodemographics and barriers: selecting plant-based foods

In everyday life, food selection is often a routinized process. However, many economic, environmental, social, psychological and cultural factors are involved in why people eat the way they do (12). If one is accustomed to consuming meat at most meals, it may be difficult to alter such routinized ways of eating. Furthermore, a variety of societal and cultural practices maintain a meat-eating culture. For instance, meat is readily available at grocery stores, and restaurants are usually assumed to offer meat dishes, unless they are profiled as vegetarian or vegan. Furthermore, liking the taste of meat, the price of meat alternatives and the routinized nature of meat eating are significant barriers for reducing meat consumption (13). Skórska et al. (14) discovered that as a large part of Western cooking recipes center around meat, consumers are steered towards meat instead of plant-based protein sources.

Before and in the beginning of the 20th century, meat consumption in Finland was quite scarce, and pulses together with grains were an important source of protein (15, 16). After World War II, meat has had an important role in the Finnish food culture with most people consuming it regularly [e.g., (17)], and a significant amount of the Finnish population consume red and processed meat more than the national nutrition guidelines recommend (9). However, in recent years the consumption of all types of meat (beef, pork, poultry, game) has been decreasing slowly (18). The recently published Nordic Nutrition Recommendations 2023 advocate more plant-based diets by decreasing the amount of meat and increasing the consumption of pulses (19).

As meat consumption has already been decreasing and more plant-based diets are called for, it is an opportune time to try to quench the cultural appeal of meat by replacing it with pulse and other plant-based alternatives. Soy protein granules and tofu may be the most traditional and well-known meat alternatives, but in recent years different plant-based patties, sausages as well as various other forms of meat-like products have fast gained shelf-space in supermarkets (20). Such new products often imitate meat in sensory properties, like mouthfeel and taste (21), which often helps consumers to accept them as they provide a familiar alternative. In this paper we use the abbreviation “PBMA” to refer to all (processed) plant-based meat alternatives. The concepts of “plant protein food” and “plant-based food” include the afore-mentioned PBMAs as well as all other kinds of plant protein sources, such as fresh, dried and canned beans and peas.

Earlier studies have found typical pulse consumers to be young and women with a higher education, interested in healthy eating and living in large or medium-sized cities (14, 22, 23), and more sustainable eaters to be women, young, and highly educated (24). Furthermore, current consumers of pulses and pulse-based meat alternatives are also interested in consuming them more in the future (25, 26). Men, people with higher income, lower education level and disinterest in healthy eating gravitate more towards animal protein source consumption (13, 22, 23, 27, 28).

Major reasons for not consuming pulses are not being used to eating them, not knowing recipes for preparing them and not being interested in them (29). Additional barriers to pulse consumption found are, i.e., unpleasant gut symptoms (29), inconvenience and long preparation times (28, 30), inability to cook pulses and scarce number of traditional meals containing them (14) and the unpleasant taste of beans (25, 29, 31). Mäkiniemi and Vainio (32) found that price was the most decisive barrier to climate-friendly food selection, including increasing plant protein food consumption. However, there is as of yet little research on the association between sociodemographic factors and pulse consumption barriers [see (33)]. Our paper examines this relationship.

### 2.2. The COM-B model and plant-based eating

The COM-B model, developed by Michie, van Stralen, and West (11), provides a fruitful theoretic background for examining different habits, such as exercising, smoking and, as in this paper, eating. Furthermore, the COM-B model forms the center of the Behaviour Change Wheel (BCW) (11), which enables the planning of effective behavior change interventions. The COM-B model is a rather simple yet comprehensive system to analyze the different capabilities (C), opportunities (O) and motivations (M) people have for acting the way they do, and how these three components in turn may influence (and be influenced by) actual behavior (B). The COM-B model and BCW are effective in identifying how and which components should be transformed in order to achieve the desired behavior change. All three COM factors are required to be present for a behavior to occur, and for a behavior to change, as the factors interact with each other (11). As an example, if one enjoys plant-based foods and is thus motivated to consume them, but lacks the skills to cook such dishes and at the same time the selection of products is narrow or non-existent meaning that capability and opportunity are absent, one is not very likely to commit to a plant-based diet and motivation to do so may decrease as well [see (34)].

Michie et al. (11) define all three COM-B factors as comprising of two dimensions. Capability is divided into *physical and psychological capability*. In the context of eating, physical capability may refer to the bodily ability to cook, and psychological capability being able to understand recipes and how to modify them. Opportunity consists of *social opportunity*, which can be defined as the surrounding social environment, and the *physical opportunity* concerning which food items are on offer and their affordability. Motivation includes *reflective motivation* such as planning what will be eaten and for what reasons (e.g., health, environment), and *automatic motivation* that takes into account emotions and impulses, like smelling something good and suddenly wanting to eat that even though something else was previously planned – thus overriding the reflective motivation.

The BCW, and the COM-B model in its center, offers a suitable framework for planning (eating) behavior change interventions, since it takes into account both the inner and outer behavior cues. The intervention measures presented in the BCW are closely linked with the three elements of the COM-B, and thus with the BCW it is possible to target the COM-B factors appropriately when mapping out interventions. Atkins and Michie (35) note that behavior is contextual and materializes within a system of behaviors, which occurs at different levels. Eating is often habitual behavior, which is heavily influenced by the environment (36), i.e., the social and physical opportunities. West and Michie (34) point out that capability and opportunity affect motivation, which then may influence the actual behavior, but that behavioral motivation also competes with alternate behaviors. This means that sometimes it is necessary to decrease another behavioral motivation in order to achieve the targeted behavior (34). Hence, in the context of more plant-based eating, it might be worthwhile to try to decrease the motivation to eat meat, while strengthening the motivation to switch to a more plant-based diets, as well as to try to modify the environment which facilitates habitual (meat) eating behavior.

Graça et al. (37) employed the COM-B in their analysis of consumption orientations and the willingness to transition towards more plant-based diets. They noted that all three components needed improving in order to increase plant-based eating, as did van den Berg et al. (13). Social opportunity was lacking, as social image was seen as a barrier to eat more plant-based meals, and led to abandoning capability and motivation needed for this (37). Consumers who expected eating-related pleasure needed motivation enhancing in relation to better taste and expectations towards plant-based meals (37). Van den Berg et al. (13) found that concerning meat consumption decrease, motivation and opportunity were lacking the most, as the taste of meat and perceived high prices of meat alternatives were seen as barriers.

In a systematic review comprising over one hundred studies, Graça et al. (38) examined reducing meat consumption and following plant-based diets by employing the COM-B model. They noted that while the volume of research on motivational factors regarding more plant-based eating is ample and increasing, studies including opportunity and capability variables are lacking. Our paper focuses on each aspect of the COM-B, and thus allows a broader insight into the factors affecting the consumption of pulses and other plant-based foods. In addition, we analyze the capabilities, opportunities and motivations related to plant protein consumption barriers by taking into account the different sociodemographic factors as well. This helps to identify whether different sociodemographic groups would benefit from varying kinds of actions when advancing more plant-based food consumption.

## 3. Materials and methods

### 3.1. Participants and data collection

The consumer survey was part of the Leg4Life (Legumes for sustainable food system and healthy life) research project funded by the Strategic Research Council at the Academy of Finland. The survey was conducted in Finnish and the aim was to collect data on how consumers perceive pulses and PBMAs, their current and future use as well as barriers and enablers related to pulse consumption. The survey data was collected online in September–October 2020 by a consumer and market research company Makery Oy via their existing consumer panel in Finland. The sample (*n* = 1,000) consisted of 18–75 year-old women and men, and was stratified by gender, age group, education level and residential area. Comparison of the participants’ sociodemographic characteristics with the Finnish national statistics indicated that the sample represented the Finnish adult population well (for details, see Table 1). The participants did not receive monetary compensation for their contribution, but earned points which can be used to buy goods from the panel’s web shop or be donated to charity.

**Table 1:** Descriptive characteristics of the participants, *n* = 1,000.

	*N*	%	Comparison with Finnish population, %*
**Gender**			
Men	500	50	50.2
Women	500	50	49.8
**Age group**			
18–34 years	293	29.3	28.3
35–54 years	352	35.2	34.4
55–75 years	355	35.5	37.3
**Residential region**			(Continental Finland)
Helsinki and Uusimaa region	311	31.1	31.5
Southern Finland (excluding Helsinki and Uusimaa)	200	20.0	20.9
Western Finland	259	25.9	24.8
Eastern and Northern Finland	230	23.0	22.8
**Urban–rural residence**			
Rural area	214	21.4	
Urban area	786	78.6	
**Education level**			(20-74-year-olds)
Tertiary	358	35.8	36.7
Secondary	509	50.9	46.1
Elementary	133	13.3	17.2
**Perceived financial situation**			
No financial strain	442	44.2	
Ok when frugal	375	37.5	
Financial strain	183	18.3	
**Current consumption of pulses**			
Never	78	7.8	
Less than once a month	215	21.5	
1–3 times a month	284	28.4	
Once a week	192	19.2	
2–4 times a week	182	18.2	
5–6 times a week	34	3.4	
Daily	15	1.5	
Current consumption of PBMAs**			
Never	455	45.5	
Less than once a month	255	25.5	
1–3 times a month	130	13.0	
Once a week	75	7.5	
2–4 times a week	66	6.6	
5–6 times a week	12	1.2	
Daily	7	0.7	
**Future consumption of pulses**			
Increases	260	26.0	
Stays the same/decreases/do not know	740	74.0	
**Future consumption of PBMAs**			
Increases	195	19.5	
Stays the same/decreases/do not know	805	80.5	
**Perceived barriers**			
Products are not familiar	440	44.0	
Expensive price	437	43.7	
Do not like the taste	411	41.1	
Unpleasant mouthfeel	337	33.7	
Do not know how to prepare [pulses]	311	31.1	
Family does not want to eat [pulses]	304	30.4	
Narrow product selection	237	23.7	
Do not suit me (e.g., cause stomach problems)	224	22.4	
Preparing [pulses] is tedious	218	21.8	
Hard to find in a store	202	20.2	

*Source: Statistics Finland (2021) and **Plant-based meat alternatives.

#### 3.1.1. Ethical issues

Prior to data collection, the survey and its protocol were reviewed by the University of Helsinki Ethical Review Board in Humanities and Social and Behavioral Sciences (Statement 40/2020). The respondents were provided detailed information on the study beforehand and they gave their informed consent electronically.

### 3.2. Measures

The sociodemographic factors examined included gender, age, residential region, urban–rural residency, education, and perceived financial situation. The response options for gender were woman, man and other, but none of the participants chose the last option. Age was recoded into three age groups (18–34, 35–54, and 55–75 years). Participants’ place of residence was recoded into four geographical regional categories: Helsinki and Uusimaa region, Southern Finland, Western Finland, and Eastern and Northern Finland. Despite being geographically part of Southern Finland, Helsinki and Uusimaa region was separated into its own category, as Helsinki is the capital of Finland and around 30% of the Finnish population resides in Helsinki and Uusimaa region. Participants’ place of residence was also categorized into urban or rural region as defined by Statistic Finland (39). Education was recoded into three classes: elementary, secondary and tertiary education, in which all higher education levels (bachelor, master and post-graduate) were combined into tertiary education. The participants were asked to estimate their own financial situation on a 5-point scale: I get by excellently, I get by quite well, I get by when I shop frugally, I sometimes need to compromise and I need to compromise almost all the time. The first two response options were recoded into a combined category “no financial strain” and the last two options into “financial strain” category, thus resulting in a three-category perceived financial situation variable.

Current pulse and PBMA consumption were studied with the question “How often do you consume the following foods?” with choices for (a) beans, lentils and peas and (b) PBMAs (examples with familiar Finnish product names given in brackets). The response options were never, less than once a month, 1–3 times a month, once a week, 2–4 times a week, 5–6 times a week and daily. These response options were recoded into a dichotomous scale as follows: 1 = never or less than once a month and 0 = more often than once a month for pulse consumption and 1 = never and 0 = at least sometimes for PBMA consumption. This difference in recoding was due to the different share of non-consumers: 46% of the participants reported never consuming PBMAs, while the respective proportion was 8% for pulses.

Future pulse and PBMA consumption were investigated with the question “How do you believe your consumption of the following foods will change in the near future?” (in this paper, we refer to this as future consumption) with choices for (a) beans, lentils and peas and (b) PBMAs (examples with familiar product names given in brackets). The response options (decreases, stays the same, increases and do not know) were recoded into the following dichotomous scale: 1 = increases and 0 = other.

The participants were also asked to evaluate to which extent ten different factors limit their use of pulses and pulse-based products (the two food item types were combined in the same original variable). These factors (later: barriers, see Table 1) were measured on a 5-point Likert scale (1 = does not limit at all – 5 = limits significantly) with “do not know” as an additional response option. The measured barriers were recoded into dichotomous variables as well. Responses with values 1–3 and “do not know” were recoded into “0 = not a barrier” and responses with values 4–5 were recoded into “1 = barrier.”

### 3.3. Statistical analyses

The relationships between current and future consumption of pulses and PBMAs, sociodemographic factors and perceived barriers were analyzed with logistic regression using IBM SPSS Statistics version 26 (IBM Corp., Armonk, NY, United States). The results are reported as odds ratios (ORs) and 95% confidence intervals (CIs). The threshold for statistical significance was set at *p* < 0.05.

Four binary logistic regression models were conducted with sociodemographic and dichotomous barrier variables predicting the odds of (1) using pulses less than once a month or never (29% of participants), (2) never using PBMAs (46%), (3) increased future pulse use (26%), and (4) increased future PBMA use (20%). All predictors were entered simultaneously into the models (see Appendix Table A.1 for logistic regressions testing bivariate associations between each predictor and outcome).

The reference category for each sociodemographic predictor was chosen based on prior studies’ results on who are the most likely users of pulses and/or (other) plant proteins. The chosen reference categories were: female, youngest age group of 18–34 years, Helsinki and Uusimaa region (large and medium-sized, urban cities), tertiary education and no financial strain. Reference category for the perceived barriers was “no barrier.”

To ensure the suitability of including all sociodemographic and barrier variables as predictors into the same model, Spearman correlation tests were carried out beforehand. All correlations were below 0.5 suggesting no substantial multicollinearity between the predictors.

Ten binary logistic regression models were then performed with sociodemographic factors predicting the odds of each barrier. Again, all predictors were entered simultaneously into the models (see Appendix Tables B.2A,B for logistic regressions testing bivariate associations between each predictor and outcome).

Finally, the number of reported barriers and their association with sociodemographic factors was tested with multinomial logistic regression. The odds of reporting “zero barriers” or “six or more barriers” (with “one to five barriers” as a reference category) was predicted by gender, age group, education level, perceived income and residential region.

## 4. Results

### 4.1. Current pulse and PBMA consumption in relation to sociodemographic factors and perceived barriers

We examined 10 perceived barriers often associated with consuming plant-based foods (Table 1). All barriers had prominence at some level, but the most common barriers related to eating pulses were unfamiliarity (44%), expensive price (44%), and not liking their taste (41%). Barriers limiting pulse consumption the least were finding cooking them tedious (22%) and experiencing them hard to find in a store (20%).

Table 2 (left-hand side) shows that the respondents’ current consumption of pulses and PBMAs varied according to their sociodemographic characteristics. Lower than tertiary education and financial strain indicated less likely consumption of pulses and PBMAs. Furthermore, age and gender were related to PBMA consumption: men and respondents over 35 years old had lower odds to consume PBMAs than women and the youngest age group.

**Table 2:** Estimates from multivariable logistic regression models: sociodemographic factors and perceived barriers predicting current and future consumption of pulses and plant-based meat alternatives (PBMAs), *n* = 1,000.

	Current use of pulses (less than once a month or never)		Current use of PBMAs (never)		Future use of pulses (increases)		Future use of PBMAs (increases)	
	OR	95% CI	OR	95% CI	OR	95% CI	OR	95% CI
**Gender**								
Women	1		1		1		1	
Men	1.08	(0.81–1.45)	1.37*	(1.04–1.80)	0.60**	(0.45–0.81)	0.72	(0.52–1.01)
**Age**								
18–34 years	1		1		1		1	
35–54 years	0.77	(0.53–1.10)	1.58*	(1.12–2.24)	0.80	(0.55–1.16)	0.78	(0.52–1.15)
55–75 years	0.76	(0.53–1.09)	2.74***	(1.94–3.86)	0.87	(0.61–1.26)	0.46***	(0.30–0.70)
**Region**								
Helsinki and Uusimaa	1		1		1		1	
Southern Finland	1.13	(0.74–1.72)	1.25	(0.85–1.84)	1.01	(0.67–1.52)	0.97	(0.61–1.54)
Western Finland	1.35	(0.91–1.99)	1.14	(0.79–1.64)	0.65*	(0.43–0.98)	0.80	(0.51–1.25)
Eastern and Northern Finland	1.44	(0.96–2.14)	1.17	(0.80–1.70)	0.99	(0.67–1.48)	0.92	(0.59–1.45)
**Urban–rural residence**								
Urban area	1		1		1		1	
Rural area	1.15	(0.81–1.64)	1.29	(0.91–1.81)	0.67	(0.45–1.07)	0.60*	(0.37–0.95)
**Education level**								
Tertiary	1		1		1		1	
Secondary	1.55**	(1.11–2.16)	1.96***	(1.45–2.65)	0.69*	(0.50–0.95)	0.68*	(0.48–0.98)
Elementary	2.19**	(1.37–3.48)	2.51***	(1.60–3.93)	0.46**	(0.27–0.78)	0.77	(0.44–1.35)
**Perceived financial situation**								
No financial strain	1		1		1		1	
Ok when frugal	1.16	(0.83–1.60)	1.21	(0.89–1.64)	1.18	(0.84–1.64)	0.85	(0.59–1.23)
**Financial strain**	1.89**	(1.27–2.81)	1.69**	(1.15–2.50)	1.04	(0.68–1.60)	0.59*	(0.36–0.98)
**Barriers**								
*Not familiar*								
No barrier	1		1		1		1	
Barrier	1.94***	(1.39–2.69)	1.52**	(1.11–2.08)	0.91	(0.64–1.28)	1.24	(0.85–1.81)
*Expensive price*								
No barrier	1		1		1		1	
Barrier	0.74	(0.54–1.01)	0.49***	(0.36–0.66)	1.11	(0.80–1.53)	1.22	(0.86–1.75)
*Do not like the taste*								
No barrier	1		1		1		1	
Barrier	1.97**	(1.33–2.90)	1.73**	(1.18–2.52)	0.50**	(0.32–0.76)	0.57*	(0.36–0.91)
*Unpleasant mouthfeel*								
No barrier	1		1		1		1	
Barrier	0.84	(0.56–1.25)	1.02	(0.69–1.50)	1.37	(0.89–2.13)	0.91	(0.56–1.47)
*Do not know how to prepare them*								
No barrier	1		1		1		1	
Barrier	1.06	(0.73–1.55)	0.96	(0.67–1.37)	1.13	(0.77–1.67)	1.94**	(1.28–2.94)
*Family does not want to eat them*								
No barrier	1		1		1		1	
Barrier	0.90	(0.63–1.28)	1.02	(0.73–1.43)	1.33	(0.92–1.91)	1.03	(0.69–1.55)
*Narrow product selection*								
No barrier	1		1		1		1	
Barrier	1.01	(0.68–1.51)	1.13	(0.77–1.65)	1.07	(0.72–1.60)	0.89	(0.57–1.40)
*Do not suit me (e.g., cause stomach problems)*								
No barrier	1		1		1		1	
Barrier	1.00	(0.69–1.44)	1.15	(0.81–1.63)	0.63	(0.47–1.02)	0.59*	(0.37–0.93)
*Preparing them is tedious*								
No barrier	1		1		1		1	
Barrier	0.86	(0.57–1.30)	0.92	(0.62–1.37)	0.90	(0.59–1.38)	0.67	(0.41–1.07)
*Hard to find in a store*								
No barrier	1		1		1		1	
Barrier	0.57*	(0.37–0.88)	0.48***	(0.32–0.72)	1.80**	(1.19–2.71)	2.06**	(1.31–3.24)

***p < 0.001, **p < 0.01, and *p < 0.05.

Our analysis of the association between pulse and PBMA consumption and perceived barriers showed that unpleasant taste and unfamiliarity with the foods resulted in their less likely consumption (Table 2, left-hand side). In addition, expensive price and the difficulty of finding the products in a store were linked to PBMA consumption: respondents finding expensive price and not being able to find the products in a store to be barriers were more likely to consume PBMA than those who did not perceive these as barriers.

### 4.2. Future pulse and PBMA consumption in relation to sociodemographic factors and perceived barriers

In future consumption of pulses and PBMAs, the sociodemographic trends were largely similar to those detected above for current consumption (Table 2, right-hand side). Having a lower than tertiary education indicated lower odds for increasing pulse consumption, and financial strain was associated with less likely increase in PBMA use.

Increase in men’s pulse consumption was less likely than women’s, and the oldest respondents had lower odds of increasing their PBMA consumption than the youngest ones. In addition, respondents living in a rural area had lower odds of increasing their PBMA use.

The perceived barriers associated with future pulse consumption were not liking the taste and experiencing pulse products hard to find in a store. Not liking the taste of pulses was related to lower odds of increasing their use. Conversely, experiencing pulses and pulse-based products hard to find in a store led to higher odds of future increase in pulse consumption.

Experiencing pulses and pulse-based products unsuitable (e.g., unpleasant gut symptoms) was linked to lower odds for increasing PBMA use in the future. Respondents perceiving pulses and pulse-based products hard to find in a store and not knowing how to cook them as barriers were more likely to increase their PBMA use.

### 4.3. Sociodemographic factors predicting perceived barriers

The results of the analysis on sociodemographic factors predicting perceived barriers are presented in Tables 3, 4.

**Table 3:** Estimates from multivariable logistic regression models: sociodemographic factors predicting perceived barriers (*n* = 1,000).

	Not familiar		Expensive price		Do not like the taste		Unpleasant mouthfeel		Do not know how to prepare [pulses]	
	OR	95% CI	OR	95% CI	OR	95% CI	OR.	95% CI	OR	95% CI
**Gender**										
Women	1		1		1		1		1	
Men	0.72*	(0.56–0.93)	1.03	(0.80–1.34)	0.97	(0.75–1.25)	0.99	(0.76–1.29)	0.70*	(0.53–0.92)
**Age**										
18–34 years	1		1		1		1		1	
35–54 years	1.08	(0.78–1.49)	0.89	(0.64–1.23)	0.88	(0.64–1.21)	0.95	(0.68–1.33)	0.90	(0.64–1.26)
55–75 years	1.03	(0.75–1.41)	0.71*	(0.51–0.97)	0.66*	(0.48–0.91)	0.88	(0.63–1.23)	0.67*	(0.47–0.94)
**Region**										
Helsinki and Uusimaa	1		1		1		1		1	
Southern Finland	1.04	(0.72–1.51)	0.83	(0.57–1.20)	0.89	(0.62–1.29)	0.98	(0.66–1.43)	0.74	(0.50–1.11)
Western Finland	1.30	(0.92–1.83)	1.01	(0.71–1.43)	1.03	(0.73–1.44)	1.18	(0.83–1.69)	0.83	(0.58–1.21)
Eastern and Northern Finland	1.37	(0.96–1.94)	1.06	(0.74–1.51)	1.14	(0.80–1.62)	1.29	(0.89–1.87)	1.22	(0.84–1.76)
**Urban–rural residence**										
Urban area	1		1		1		1		1	
Rural area	0.75	(0.54–1.03)	0.82	(0.59–1.13)	0.95	(0.69–1.31)	0.66*	(0.46–0.93)	1.03	(0.73–1.45)
**Education**										
Tertiary	1		1		1		1		1	
Secondary	1.37*	(1.03–1.82)	1.22	(0.91–1.62)	0.96	(0.72–1.27)	0.93	(0.69–1.24)	1.09	(0.80–1.47)
Elementary	1.17	(0.77–1.80)	1.17	(0.76–1.80)	0.79	(0.51–1.22)	0.66	(0.42–1.05)	0.87	(0.55–1.39)
**Perceived financial situation**										
No financial strain	1		1		1		1		1	
Ok when frugal	1.11	(0.83–1.49)	1.62**	(1.22–2.16)	1.09	(0.82–1.46)	1.20	(0.89–1.62)	1.06	(0.78–1.44)
Financial strain	1.57*	(1.09–2.24)	2.76***	(1.92–3.98)	1.29	(0.90–1.85)	1.33	(0.92–1.94)	1.33	(0.91–1.95)

***p < 0.001, **p < 0.01, and *p < 0.05.

**Table 4:** Estimates from multivariable logistic regression models: sociodemographic factors predicting perceived barriers (*n* = 1,000).

	Family does not want to eat [pulses]		Narrow product selection		Do not suit me		Preparing [pulses] is tedious		Hard to find in a store	
	OR	95% CI	OR	95% CI	OR	95% CI	OR.	95% CI	OR	95% CI
**Gender**										
Women	1		1		1		1		1	
Men	0.78	(0.59–1.02)	1.26	(0.94–1.69)	0.64**	(0.47–0.87)	1.01	(0.75–1.37)	1.09	(0.80–1.49)
**Age**										
18–34 years	1		1		1		1		1	
35–54 years	1.39	(0.99–1.95)	0.79	(0.55–1.15)	1.31	(0.89–1.91)	0.71	(0.49–1.04)	0.68	(0.46–1.00)
55–75 years	0.72	(0.51–1.03)	0.86	(0.59–1.23)	0.95	(0.65–1.41)	0.63*	(0.43–0.92)	0.73	(0.50–1.07)
**Region**										
Helsinki and Uusimaa	1		1		1		1		1	
Southern Finland	0.98	(0.65–1.46)	0.76	(0.49–1.18)	1.14	(0.75–1.73)	0.76	(0.49–1.19)	0.68	(0.42–1.09)
Western Finland	1.07	(0.74–1.55)	0.96	(0.65–1.42)	0.79	(0.53–1.20)	0.92	(0.61–1.38)	0.96	(0.63–1.45)
Eastern and Northern Finland	1.29	(0.88–1.88)	1.12	(0.75–1.68)	0.89	(0.58–1.35)	1.05	(0.69–1.58)	1.15	(0.76–1.76)
**Urban–rural residence**										
Urban area	1		1		1		1		1	
Rural area	1.28	(0.91–1.80)	0.85	(0.58–1.25)	1.16	(0.79–1.68)	0.95	(0.65–1.40)	0.98	(0.66–1.46)
**Education**										
Tertiary	1		1		1		1		1	
Secondary	1.21	(0.90–1.65)	1.10	(0.79–1.53)	0.89	(0.64–1.24)	0.98	(0.70–1.37)	1.09	(0.77–1.55)
Elementary	0.89	(0.55–1.43)	0.79	(0.47–1.32)	0.82	(0.49–1.36)	0.83	(0.49–1.40)	0.78	(0.45–1.35)
**Perceived financial situation**										
No financial strain	1		1		1		1		1	
Ok when frugal	0.86	(0.63–1.17)	1.06	(0.76–1.49)	1.43*	(1.02–2.02)	1.06	(0.75–1.49)	0.88	(0.62–1.26)
Financial strain	1.07	(0.73–1.57)	1.45	(0.97–2.18)	1.43	(0.94–2.18)	1.53*	(1.01–2.31)	1.31	(0.85–2.01)

***p < 0.001, **p < 0.01, and *p < 0.05.

*Gender* Men had lower odds of finding not being familiar with pulses being a barrier for pulse consumption than women did. Men also found not knowing how to cook pulses or pulses not suiting them to be less of a barrier than these were to women.

*Age* Compared to the youngest age group, the oldest age group had lower odds of perceiving pulses’ expensive price a barrier. The oldest respondents were also less likely to find not liking the taste of pulses to be a barrier than the youngest age group. In addition, the oldest respondents had lower odds of not knowing how to cook pulses and finding cooking pulses to be tedious than the youngest participants.

*Education and perceived financial situation* Respondents with secondary education had higher odds of experiencing not being familiar with pulses a barrier compared to respondents with tertiary education. Financially strained respondents and respondents getting by when shopping frugally were more likely to find pulse-based products’ expensive price a barrier than respondents with no such strain. Being financially strained was also related to experiencing more unfamiliarity with pulse-based products and finding cooking them tedious.

*Number of reported barriers* Altogether 19.2% reported 0 barriers and 18% reported 6 or more barriers (no table). The results from multinomial logistic regression revealed that financial strain was associated with reporting more barriers. Compared to participants with financial strain, those with no such strain had 1.7-fold odds of reporting 0 barriers (95% CI 1.03–2.90, *p* < 0.05) and 0.6-fold odds of reporting 6 or more barriers (95% CI 0.38–0.96, *p* < 0.05). Other sociodemographic factors were not significantly related to the number of reported barriers.

## 5. Discussion

We first discuss the general findings of the study. Thereafter we reflect on the findings by employing the COM-B model in analyzing the perceived barriers to pulse and other PBMA consumption. In addition, we take into account the role of sociodemographic factors in our analysis. Lastly, we consider possible measures for future interventions to increase pulse and PBMA consumption.

### 5.1. General findings

Our results show that pulses are consumed more often than PBMAs: over 90% of the respondents consumed pulses at least sometimes, while just over half of the respondents consumed PBMAs. In total about one fourth of the respondents thought of increasing their pulse consumption, while roughly one fifth planned to increase their PBMA consumption. The most significant barriers for pulse consumption were unfamiliarity (44%), expensive price (44%), and unpleasant taste (41%) of pulses. These barriers represent rather well how all of the COM-B aspects affect the conditions of plant-based eating.

Male gender and older age was associated with lower current PBMA consumption. In addition, men had less intentions to increase their pulse consumption, and older respondents were not likely to increase their PBMA consumption. Siegrist and Hartmann (40) found that women are more likely consumers of meat alternatives, and Jallinoja et al. (25) that women consume beans more often than men. Graça et al. (38) point out that findings from various studies show that the male gender is often associated with unwillingness and the female gender with willingness to follow plant-based diets, but that results concerning age are mixed. Interestingly in our study, men and older respondents found certain barriers to limit their pulse consumption less than women and the young did, even though the latter are more often profiled to be more interested in plant-based foods.

Earlier research shows that higher education predicts more frequent bean consumption (25), and readiness to accept PBMAs (40, 41). Overall, higher socioeconomic status acts as an enabler to consume plant-based foods (38), and different economic situation may lead to different access to certain consumer goods (42). Our results are in line with this, as lower education level and financial strain were associated with lower pulse and PBMA consumption. Living area was not strongly associated with pulse and PBMA consumption, even though some earlier research has found that living in large or medium-sized cities often indicates a tendency to consume plant-based foods [e.g., (14)].

### 5.2. Capabilities, opportunities, and motivations limiting the consumption of pulses and pulse-based products

#### 5.2.1. Unfamiliarity and preparation capabilities

Being unfamiliar with pulses and other plant-based foods is a significant barrier for their consumption, as earlier research [e.g., (29)] as well as our results show. Unfamiliarity affects the *psychological capability* to eat these kinds of foods. We discerned that current use of both pulses and PBMA products was likely to be limited due to unfamiliarity. In addition, some sociodemographic groups found unfamiliarity to be more of a barrier than others; most notably women and those with financial strain. We propose that the gender difference may be because some men are not that interested in consuming pulses or PBMAs, and thus do not experience any barriers related to them either. Furthermore, if money is tight, it may feel safer to buy familiar options to get one’s money’s worth (43, 44). Oude Groeniger et al. (45) argue that consuming healthy food is one mechanism for social distinction in the higher socioeconomic classes, and Bowman (46) points out that lower education level can in some cases be associated with the lack of knowledge of the benefits of more plant-based eating, and that plant-based eating might be seen as something that belongs to those with higher education (and thus often higher socioeconomic status). To summarize, pulse unfamiliarity as a psychological capability affects especially people with lower socioeconomic status and women.

Unfamiliarity is closely related to inability to cook pulses and pulse-based products and finding preparing them to be tedious, which can be classified as both *psychological* (e.g., not knowing how) and *physical* (e.g., not having enough time) *capability*. Nguyen et al. (47) conceptualize cooking skills as a physical capability, and their absence acts as a barrier to alternative protein source consumption. Again, women experienced the inability to cook pulses and pulse-based products to be more of a barrier than men did, which refers to the idea above that men may not be as interested in pulses, and thus have no barriers. However, women are more willing to eat plant-based foods (38) and to try vegetarian recipes (13) than men. Additionally, older respondents had less feelings of inability to cook pulses and pulse-based products, and they also found preparing pulses less tedious than younger respondents. We propose that this is an indication of older respondents being more used to cooking from scratch: convenience and time-saving is especially valued by the younger generation [(12); see also (48)].

#### 5.2.2. Taste and unpleasant gut symptoms

Similarly to our findings, earlier studies have concluded that the unpleasant taste of pulses is a barrier to consuming them (28, 29, 31). Furthermore, doubting the taste of PBMAs prevents their use (35), and liking meat is often a barrier for meat alternative consumption (47). In the context of eating, pleasure is one major motivation to consume certain kinds of foods (43, 49), and good taste is significant for achieving pleasure. Taste can be classified as *automatic motivation*. In our study, the unpleasant taste of pulses was a significant barrier for both current and future consumption of pulses and PBMAs. Older respondents experienced the taste to be less of a barrier than the youngest, but Jallinoja et al. (23) found that young consumers were more likely to eat beans. Thus, we argue that taste of pulses may be perceived differently by different groups of people, depending on, e.g., their generation, social upbringing and taste preferences.

Pulses are often perceived to cause unpleasant gut symptoms: even up to one third of the Western population suffers pulse digestion problems (50). This can act as a barrier for pulse use, as undesired gastrointestinal symptoms may physically limit the capability to consume pulses. Winham and Hutchins (50) found that women were more likely to report gastrointestinal symptoms and sensations, which is in line with our finding that women found pulses causing unpleasant gut symptoms more of a barrier than men did. Earlier research has also concluded that pulses’ gastrointestinal unsuitability limits the capability to consume them (14, 29, 31).

#### 5.2.3. Price

Graça et al. (38) discuss the higher price of meat as a potential enabler for increased plant protein consumption. However, there are mixed results on perceived plant protein prices: Vainio et al. (28) discovered that the expensive price of plant protein foods was the most significant barrier for their consumption, while Niva et al. (29) concluded that price was not a barrier. Furthermore, van den Berg et al. (13) found price to both prevent and enable eating PBMAs: the perceived high price of PBMAs was a barrier, but saving money was also seen as a reason to consume less meat and more plant proteins. In our study, pulses *per se* and pulse-based products were lumped together when enquiring about the price, even though their prices often differ significantly. We found that the price of pulses acted as a barrier for respondents struggling financially, thus limiting their *physical opportunity* to consume these products, which possibly indicates that they thought of pulse-based meat alternatives rather than pulses *per se*, as the latter are often quite inexpensive. Lower prices of pulse-based products would create more opportunities for consumers to increase their pulse intake (30). We also found that those already consuming PBMAs perceived their price to be more of a barrier than non-consumers. We propose two explanations for this. First, there may be a group of non-consumers for whom the barrier is not the price of the products, but other factors. Secondly, there may be consumer groups, such as young students, who would like to consume more PBMAs, but find their high price to limit their consumption opportunities.

Age can also be a factor affecting the perception of price: we found that the oldest respondents had less of a price barrier than the youngest, which suggests that they may be financially better off or that they may have thought of different pulse products, i.e., pulses *per se* versus processed products. Our notion of older respondents not consuming and not intending to consume PBMAs supports this interpretation. In addition, perceptions of plant-based foods and their price may vary culturally: the Spanish found plant-based eating more affordable than the Danish (51). In Spain, the traditional Mediterranean diet includes affordable dried and fresh pulses (52), while Northern Europeans often like to emphasize the convenience of food products (53), and thus may prefer more expensive, ready-to-use pulse products.

#### 5.2.4. Store and family environment

Another physical opportunity affecting pulse and PBMA consumption was having difficulties in locating these products in a store. Earlier research has noted that more prominent positioning of healthy foods (such as pulses) in grocery stores is related to healthier food choices (54) and that alternative protein foods are often placed separately from meat equivalents (55). Thus, with better placement of plant protein foods, grocery stores could advance their consumption. Curiously, respondents already consuming pulses and PBMAs and intending to increase their consumption found not being able to find these products in a store to be more of a barrier than non-consumers. Again, there might be a group of non-consumers who have not even tried to find pulses in a store, which is why it is not a barrier. Other explanations can also be offered. The first is that some stores may not carry a large selection of pulse products, which is why they are hard to find (however, this did not seem to be an issue when examining “narrow product selection” barrier). The second is that some products might be stocked low and often sold out, resulting in not finding them. The third is that not all stores have clearly organised sections where all pulse products are conveniently on display, resulting in shoppers needing to navigate through multiple sections before finding the right product (or not finding it at all), which also calls for their better positioning.

Social environment can act either as barrier or enabler for decreasing meat consumption and/or increasing the portion of plant-based foods (38). We only examined family as the social environment (i.e., social opportunity) relating to pulse consumption barriers, and even though 30% of respondents perceived this as a barrier, it was not statistically significant in our study. Van den Berg et al. (13) had similar results, as they noted that people were mostly able to decide themselves which foods to consume regardless of the social environment. Other barriers which were not statistically significant in relation to current and future pulse and PBMA consumption in our study were mouthfeel (relating to the pleasure aspect of motivation) and narrow selection of pulse products (physical opportunity).

#### 5.2.5. Intervention measure suggestions

Habits are often unconscious and triggered by the environment, and intentions do not necessarily translate into actual behavior (36). One behavior may be a barrier to another (34), and automatic motivation may override reflective motivation (35). Thus, to conclude our discussion, we propose some possible future intervention measures within the COM-B model to increase the consumption of pulses and PBMAs.

As the present results demonstrate, lower education level and financial struggle are associated with consuming pulses and PBMAs more infrequently. One commonly proposed solution is to increase the proportion of plant-based meals in workplace canteens, but the problem is that people with lower socioeconomic status often do not have access to these places (56) and consume homemade or bought food. However, in Finland, children are provided with free daycare and school lunches (57), and thus one way to increase plant-based eating could start from these public food services. There have been prior attempts to increase the amount of vegetarian food in schools that have led to mixed results: on the other hand, food waste increased and participation at lunch decreased, but some vegetarian dishes gained popularity (58). To further increase plant-based food consumption at school lunches, the physical opportunities need to be taken into account. One way to enhance this is to place plant-based dishes first on the serving line. However, for plant-based dishes to be more readily accepted, the social opportunities need to be present as well. At present, meat dishes are seen as more “normal,” as the need to specifically distinguish vegetarian food days in schools demonstrates: there is no talk of “meat food days.” Thus, plant-based foods should be normalized and not differentiate them from meat dishes conspicuously. In addition, home economics classes at schools can help to normalize plant-based foods and to achieve capabilities to cook tasty and nutritious plant-based meals. All these measures would also make pulses more familiar, which is an enabler for their consumption (10).

The present findings suggest that the food industry and retail trade can enhance the motivation and physical opportunities to eat plant protein foods. We noted that unpleasant taste was one significant barrier for consuming pulses and PBMAs. Improving taste qualities of plant protein foods and maybe offering appealing convenience meals without meat could increase the motivation to consume them. Furthermore, perceived high prices hindered plant protein food consumption. To steer consumers towards plant protein foods, changes in taxing meat and plant protein sources could make plant foods more appealing and financially more available to all. Finally, grocery store settings could be modified to help consumers find meat alternatives without having to make a significant effort. For example, Piernas et al. (59) found that by placing alternative proteins in the meat aisle, their sales increased. Thus, by placing plant proteins next to meat products would make it easier for consumers to find and buy meatless alternatives.

### 5.3. Strengths and limitations

One strength of the study lies in using large and recent survey data including respondents who relatively well represent the general Finnish adult population in terms of gender, age, living area and education level. The most notable strength is that we analyzed the role of sociodemographic factors simultaneously with the consumption of pulses and PBMAs and the barriers related to them; a topic that is of importance if a transition towards more plant-based food consumption is to be achieved.

One limitation to consider is that pulses as raw ingredients and pulses as processed products were lumped together in the survey when enquiring about the factors perceived as barriers to their use. This is somewhat problematic, because pulses *per se* and processed pulse products often differ in price, preparation technique and taste, amongst other things. Furthermore, there is no certainty that the self-reported pulse consumption reflects actual consumption, or that intentions to increase consumption will actually be realized.

## 6. Conclusion

Our results show that the level of engagement among Finnish adults in the consumption of plant-based protein foods is currently not very high, and the intentions to increase their consumption are rather low as well. As our study demonstrates, sociodemographic factors have a role in pulse and PBMA consumption, most notably gender, age, education and perceived financial situation. Age and gender were also prominent factors in relation to perceived barriers to pulse and pulse-based product consumption. Our research also shows that the COM-B model is relevant when examining pulse and PBMA consumption. First, eating plant-based foods requires motivation to do so, and the major motivational barrier in our study was the unpleasant taste. Second, there needs to be suitable opportunities to further engage consumers in pulse and other plant-based protein consumption, e.g., in terms of affordability and store settings. Third, consumers need sufficient capabilities to be able to prepare enticing pulse- or other plant-based dishes, but unfamiliarity with and uncertainty in preparing pulses and PBMAs are barriers to their consumption. Thus, offering easy to use, tasty and affordable pulse and other plant protein-based dishes and products can pave the way towards more environmentally sustainable food consumption.

## Data availability statement

The raw data supporting the conclusions of this article will be made available by the authors, without undue reservation.

## Ethics statement

The studies involving humans were approved by University of Helsinki Ethical Review Board in Humanities and Social and Behavioral Sciences. The studies were conducted in accordance with the local legislation and institutional requirements. The participants provided their consent electronically.

## Author contributions

SK, MN, A-MP, KK, TM, and HK contributed to conception and design of the study. HK, KK, A-MP, and TM were responsible for data collection. SK performed statistical analyses, wrote the first draft of the manuscript, and had primary responsibility for the final content of the manuscript. HK collaborated in conducting the statistical analyses. HK, MN, A-MP, KK, and TM commented and critically revised the manuscript. All authors contributed to the article and approved the submitted version.

## Funding

This research was funded by the Strategic Research Council at the Academy of Finland (grants 327698 and 327700), the Academy of Finland (grants 309157 and 314135 to HK and grant 315898 to MN), Wihuri Foundation (grant 00210156 to SK) and Raisio Plc’s Research Foundation (personal grant to SK). The funding sources had no involvement in study design, data collection, analysis or interpretation, writing the article, or in the decision to submit the article for publication.

## Conflict of interest

The authors declare that the research was conducted in the absence of any commercial or financial relationships that could be construed as a potential conflict of interest.

## Publisher’s note

All claims expressed in this article are solely those of the authors and do not necessarily represent those of their affiliated organizations, or those of the publisher, the editors and the reviewers. Any product that may be evaluated in this article, or claim that may be made by its manufacturer, is not guaranteed or endorsed by the publisher.
