# Supplementary material for: Barriers associated with pulse and plant-based meat alternative consumption across sociodemographic groups: a Capability, Opportunity, Motivation, Behaviour model approach
Source: Front Nutr. 2023 Aug 29;10:1186165. doi: 10.3389/fnut.2023.1186165 (PMC10495578; doi:10.3389/fnut.2023.1186165)
Supplement: Supplementary file 1 [file Data_Sheet_1.pdf]

Supplementary Material

Barriers associated with pulse and plant-based meat alternative consumption across sociodemographic groups: a Capability, Opportunity, Motivation, Behaviour (COM-B) model approach

Sini Kuosmanen[1,2*], Mari Niva[2*], Anne-Maria Pajari[1], Kirsi Korhonen[3], Toivo Muilu[3], Hanna Konttinen[4,1]

[1]Department of Food and Nutrition, University of Helsinki, Helsinki, Finland

[2]Department of Economics and Management, University of Helsinki, Finland

[3]Natural Resources Institute Finland, Oulu, Finland

[4]Social Psychology, Faculty of Social Sciences, University of Helsinki, Helsinki, Finland

*** Correspondence:**
Sini Kuosmanen
sini.kuosmanen@helsinki.fi

1 Supplementary Data

Appendix Table A.1. Estimates from simple (bivariate) logistic regression analyses: sociodemographic factors and perceived obstacles predicting current and future consumption of legumes and plant-based meat substitutes (PBMAs), n=1000.

	Current use of legumes (less than once a month or never)		Current use of PBMAs (never)		Future use of legumes (increases)		Future use of PBMAs (increases)	
	OR	95% CI	OR	95% CI	OR	95% CI	OR	95% CI
**Gender**								
Women	1		1		1		1	
Men	0.97	(0.74-1.28)	1.19	(0.92-1.52)	0.64**	(0.48-0.86)	0.75	(0.54-1.02)
**Age**								
18-34 years	1		1		1		1	
35-54 years	0.80	(0.57-1.13)	1.57**	(1.14-2.16)	0.79	(0.56-1.13)	0.72	(0.50-1.05)
55-75 years	0.82	(0.58-1.14)	2.63***	(1.91-3.62)	0.86	(0.61-1.21)	0.48***	(0.32-0.71)
**Region**								

Helsinki and Uusimaa	1		1		1		1	
Southern Finland	1.26	(0.85-1.89)	1.32	(0.92-1.89)	0.93	(0.63-1.38)	0.91	(0.59-1.41)
Western Finland	1.43	(0.99-2.07)	1.22	(0.87-1.70)	0.62*	(0.42-0.91)	0.75	(0.49-1.14)
Eastern and Northern Finland	1.56	(1.07-2.27)	1.36	(0.96-1.91)	0.93	(0.63-1.35)	0.86	(0.56-1.32)
**Urban-rural residence**								
Urban area	1		1		1		1	
Rural area	1.30	(0.94-1.79)	1.56**	(1.15-2-12)	0.59**	(0.41-0.86)	0.53**	(0.34-0.82)
**Education level**								
Tertiary	1		1		1		1	
Secondary	1.72***	(1.26-2.35)	1.87***	(1.41-2.46)	0.74	(0.50-1.00)	0.71*	(0.51-1.00)
Elementary	2.42***	(1.57-3.71)	2.68***	(1.78-4.03)	0.50*	(0.30-0.82)	0.66	(0.39-1.10)
**Perceived financial situation**								
No financial strain	1		1		1		1	
Ok when frugal	1.25	(0.92-1.71)	1.28	(0.97-1.68)	1.03	(0.76-1.41)	0.78	(0.55-1.10)
Financial strain	2.00***	(1.39-2.89)	1.66**	(1.18-2.35)	0.93	(0.62-1.38)	0.60*	(0.38-0.96)
**Obstacles**								
Not familiar								
No obstacle	1		1		1		1	
Obstacle	1.91***	(1.45-2.51)	1.36*	(1.06-1.75)	1.01	(0.76-1.35)	1.30	(0.95-1.78)
Expensive price								
No obstacle	1		1		1		1	
Obstacle	0.91	(0.69-1.19)	0.58***	(0.45-0.75)	1.19	(0.90-1.58)	1.22	(0.89-1.67)
Don't like the taste								
No obstacle	1		1		1		1	

Obstacle	1.72***	(1.31-2.27)	1.42**	(1.10-1.83)	0.72*	(0.54-0.97)	0.68*	(0.49-0.95)
Unpleasant mouthfeel								
No obstacle	1		1		1		1	
Obstacle	1.22	(0.92-1.62)	1.17	(0.90-1.52)	1.03	(0.77-1.39)	0.80	(0.57-1.12)
Don't know how to prepare them								
No obstacle	1		1		1		1	
Obstacle	1.27	(0.95-1.70)	0.97	(0.74-1.27)	1.19	(0.88-1.60)	1.68**	(1.22-2.33)
Family doesn't want to eat them								
No obstacle	1		1		1		1	
Obstacle	1.14	(0.85-1.53)	1.07	(0.82-1.41)	1.16	(0.85-1.57)	1.05	(0.75-1.48)
Narrow product selection								
No obstacle	1		1		1		1	
Obstacle	0.99	(0.72-1.36)	0.86	(0.64-1.15)	1.26	(0.92-1.74)	1.14	(0.80-1.63)
Don't suit me (e.g. cause stomach problems)								
No obstacle	1		1		1		1	
Obstacle	1.07	(0.77-1.48)	1.13	(0.84-1.52)	0.80	(0.56-1.14)	0.63*	(0.42-0.95)
Preparing them is tedious								
No obstacle	1		1		1		1	
Obstacle	1.00	(0.72-1.40)	0.84	(0.62-1.14)	0.82	(0.74-1.46)	0.95	(0.64-1.39)
Hard to find in a store								
No obstacle	1		1		1		1	
Obstacle	0.68*	(0.48-0.98)	0.52***	(0.38-0.73)	1.70**	(1.22-2.37)	1.70**	(1.19-2.44)

*** $p < .001$, ** $p < .01$, * $p < .05$

Appendix Table B.2a. Estimates from simple (bivariate) logistic regression analysis: sociodemographic factors predicting perceived obstacles (n=1000).

	Not familiar		Expensive price		Don't like the taste		Unpleasant mouthfeel		Don't know how to prepare [pulses]	
	OR	95% CI	OR	95% CI	OR	95% CI	OR.	95% CI	OR	95% CI
**Gender**										
Women	1		1		1		1		1	
Men	0.71*	(0.55-0.91)	0.99	(0.77-1.27)	0.98	(0.76-1.26)	0.99	(0.76-1.29)	0.71*	(0.54-0.93)
**Age**										
18-34 years	1		1		1		1		1	
35-54 years	1.07	(0.78-1.46)	0.95	(0.69-1.30)	0.91	(0.67-1.25)	0.98	(0.71-1.35)	0.92	(0.66-1.28)
55-75 years	1.01	(0.74-1.38)	0.71*	(0.52-0.98)	0.66*	(0.48-0.91)	0.85	(0.61-1.18)	0.70*	(0.50-0.97)
**Region**										
Helsinki and Uusimaa	1		1		1		1		1	
Southern Finland	1.05	(0.73-1.51)	0.88	(0.62-1.27)	0.91	(0.63-1.31)	0.96	(0.65-1.40)	0.76	(0.51-1.13)
Western Finland	1.25	(0.90-1.75)	1.01	(0.73-1.41)	1.04	(0.74-1.45)	1.11	(0.78-1.57)	0.86	(0.60-1.23)
Eastern and Northern Finland	1.31	(0.93-1.84)	1.03	(0.73-1.45)	1.10	(0.76-1.55)	1.17	(0.82-1.68)	1.19	(0.83-1.71)
**Urban-rural residence**										
Urban area	1		1		1		1		1	
Rural area	0.84	(0.62-1.14)	0.88	(0.64-1.19)	0.93	(0.68-1.27)	0.68*	(0.48-0.94)	1.01	(0.73-1.40)
**Education**										
Tertiary	1		1		1		1		1	
Secondary	1.41*	(1.07-1.86)	1.36*	(1.03-1.79)	0.99	(0.76-1.31)	0.95	(0.72-1.27)	1.15	(0.86-1.53)
Elementary	1.20	(0.81-1.80)	1.28	(0.86-1.91)	0.78	(0.52-1.18)	0.65	(0.42-1.01)	0.90	(0.58-1.41)

**Perceived financial situation**										
No financial strain	1		1		1		1		1	
Ok when frugal	1.17	(0.88-1.54)	1.65***	(1.25-2.19)	1.09	(0.82-1.44)	1.15	(0.86-1.53)	1.10	(0.82-1.49)
Financial strain	1.67**	(1.18-2.37)	2.78***	(1.95-3.97)	1.25	(0.88-1.77)	1.25	(0.87-1.79)	1.34	(0.93-1.93)

*** $p < .001$, ** $p < .01$, * $p < .05$

Appendix Table B.2b. Estimates from simple (bivariate) logistic regression analysis: sociodemographic factors predicting perceived obstacles (n=1000).

	Family doesn't want to eat [pulses]		Narrow product selection		Don't suit me		Preparing [pulses] is tedious		Hard to find in a store	
	OR	95% CI	OR	95% CI	OR	95% CI	OR.	95% CI	OR	95% CI
**Gender**										
Women	1		1		1		1		1	
Men	0.81	(0.62-1.06)	1.23	(0.92-1.65)	**0.64****	(0.48-0.87)	1.00	(0.74-1.35)	1.08	(0.79-1.47)
**Age**										
18-34 years	1		1		1		1		1	
35-54 years	**1.42***	(1.02-1.98)	0.84	(0.59-1.21)	1.33	(0.92-1.92)	0.76	(0.53-1.09)	0.72	(0.49-1.06)
55-75 years	0.75	(0.53-1.06)	0.84	(0.59-1.21)	0.97	(0.66-1.42)	**0.64***	(0.44-0.93)	0.74	(0.51-1.08)
**Region**										
Helsinki and Uusimaa	1		1		1		1		1	
Southern Finland	0.99	(0.66-1.46)	0.77	(0.50-1.19)	1.14	(0.75-1.72)	0.79	(0.51-1.23)	0.69	(0.43-1.11)
Western Finland	1.17	(0.82-1.68)	0.95	(0.65-1.40)	0.83	(0.56-1.25)	0.93	(0.63-1.39)	0.97	(0.65-1.46)
Eastern and Northern Finland	1.30	(0.90-1.88)	1.09	(0.74-1.62)	0.90	(0.60-1.36)	1.01	(0.68-1.52)	1.13	(0.75-1.71)
**Urban-rural residence**										
Urban area	1		1		1		1		1	
Rural area	1.31	(0.95-1.81)	0.85	(0.59-1.23)	1.11	(0.78-1.58)	0.91	(0.63-1.32)	0.96	(0.65-1.40)
**Education**										
Tertiary	1		1		1		1		1	
Secondary	1.22	(0.91-1.64)	1.14	(0.83-1.57)	0.95	(0.69-1.31)	1.04	(0.75-1.44)	1.13	(0.81-1.59)
Elementary	0.82	(0.52-1.29)	0.82	(0.50-1.34)	0.88	(0.55-1.43)	0.87	(0.53-1.43)	0.82	(0.48-1.38)

**Perceived financial situation**										
No financial strain	1		1		1		1		1	
Ok when frugal	0.93	(0.69-1.26)	1.05	(0.75-1.45)	**1.45***	(1.04-2.02)	1.04	(0.74-1.47)	0.88	(0.62-1.25)
Financial strain	1.18	(0.82-1.70)	1.34	(0.90-1.98)	1.50	(0.99-2.25)	1.43	(0.96-2.14)	1.20	(0.80-1.82)

*** $p < .001$, ** $p < .01$, * $p < .05$
